# Calcein Binding to Assess Mineralization in Hydrogel Microspheres

**DOI:** 10.3390/polym13142274

**Published:** 2021-07-11

**Authors:** Kristopher White, Rabab Chalaby, Gina Lowe, Jacob Berlin, Carlotta Glackin, Ronke Olabisi

**Affiliations:** 1 Department of Biomedical Engineering, The Henry Samueli School of Engineering, University of California—Irvine, Irvine, CA 92697, USA; kris.white@uci.edu; 2 Department of Materials Engineering, University of Technology, Baghdad 10066, Iraq; rhhl2007@yahoo.com; 3 Department of Neuroscience, Beckman Research Institute, City of Hope, Duarte, CA 91010, USA; glowe@coh.org (G.L.); cglackin@coh.org (C.G.); 4 Department of Molecular Medicine, Beckman Research Institute, City of Hope, Duarte, CA 91010, USA; jacobberlincoh@gmail.com

**Keywords:** hydrogel, polymer, microsphere, calcein, osteogenesis, mineralization

## Abstract

We describe a method to assess mineralization by osteoblasts within microspheres using calcein. Fluorescence imaging of calcein bound to the calcium in hydroxyapatite permits assessment of the mineralized portion of the extracellular matrix. Colorimetric imaging of Alizarin Red S complexed with calcium also gives measures of mineralization, and in tissue cultures calcein and Alizarin Red S have been shown to bind to the same regions of mineral deposits. We show that when the mineralization takes place within hydrogel microspheres, Alizarin Red S does not stain mineral deposits as consistently as calcein. As tissue engineers increasingly encapsulate osteoprogenitors within hydrogel scaffolds, calcein staining may prove a more reliable method to assess this mineralization.

## 1. Introduction

The in vitro mineralization formed in osteoprogenitor cell cultures is typically assessed by von Kossa, Alizarin Red S, or, to a lesser extent, calcein staining [1,2]. All methods are used to observe the presence of calcium phosphate (hydroxyapatite) in osteoprogenitor cell cultures. In von Kossa stains, silver ions react with phosphate, forming black precipitates that can be observed with the naked eye. Alizarin Red S is considered the gold standard for assessing mineralization and it works through a chelation process in which an Alizarin Red S-calcium complex is formed, resulting in a bright red stain that can also be observed with the naked eye. Unlike the colorimetric von Kossa and Alizarin Red S stains, calcein is a fluorescent label that cannot be seen with the naked eye. However, calcein does not require fixation of cell cultures and can be used in vivo [1]. Calcein binds to the calcium in growing calcium phosphate or calcium carbonate crystals [3,4] and can be used to stain fixed or unfixed cell cultures. By itself, calcein cannot penetrate the phospholipid cell membrane. It is not to be confused with calcein AM that is used to assess cell viability. When calcein is used in such viability stains, it is complexed with an acetoxymethyl (AM) ester to form calcein AM. The AM ester eliminates calcein’s fluorescence while permitting transport across the cell membrane. When intracellular esterases cleave the AM ester, calcein becomes fluorescent again, indicating cell health. Alone, calcein exclusively binds to calcium.

Cultures can be incubated with calcein at single time points or for the duration of an experiment. Therefore, while the former methods can only assess discrete time points, continuous calcein staining permits continuous assessment of mineralization in cell cultures.

Alizarin Red S is also sensitive to pH, variations of which can result in weak to moderate stains [5]. In addition, depending on the solubility of calcium salts in the fixation solution, Alizarin Red S stains can suffer diffusion artifact. These challenges can make it difficult to assess mineralization in 2D cultures and become even more problematic in 3D cultures where the geometry of the cultures can affect the pH of the local environment and the penetration of dyes. For instance, hydrogels are increasingly being used to microencapsulate cells. In osteoprogenitor monolayers, qualitative comparisons of Alizarin Red S and calcein stain have been demonstrated to show comparable staining patterns of calcium phosphate generated by the cells [2]. In microencapsulated osteoprogenitors, we observed that this was not the case.

We compared the ability of Alizarin Red S and calcein to stain hydroxyapatite deposits formed by osteoprogenitors encapsulated within polyethylene glycol diacrylate (PEGDA) hydrogel microspheres. The von Kossa procedure was not assessed because in preliminary tests it had resulted in opaque hydrogels that had been formerly transparent. Calcein staining has repeatedly been proposed as a replacement for Alizarin Red S stain when assessing cultured osteoblasts, but it has yet to be widely adopted [1,2]. We have demonstrated that for assessing mineralization by microencapsulated osteoblasts, calcein stain is a superior method than Alizarin Red S stain.

## 2. Materials and Methods

Unless otherwise noted, all reagents used were obtained from Sigma Aldrich (St. Louis, MO, USA).

### 2.1. Cell Culture

Complete culture basal medium was prepared with Minimum Essential Media with alpha modification (α-MEM) with nucleosides supplemented with 10% Fetal Bovine Serum, 1% L-glutamine, and 1% Penicillin/Streptomycin. Passage 15 human telomerase reverse transcriptase immortalized human bone marrow mesenchymal stem cells (hTERT-hMSCs) were obtained from the Glackin Laboratory (Beckman Research Institute, City of Hope National Medical Center, Duarte, CA, USA). The osteogenic, chondrogenic, and adipogenic capacity of these cells were confirmed [6,7]. Cells isolated from 15-week human fetal bone marrow were purified according to the expression of STRO-1^bright^/CD106^+^ or STRO-1^bright^/CD146^+^, then immortalized with hTERT in a retroviral insertion vector (pBABE), then puromycin was used to select stable clones [6,7]. Resulting hTERT-MSCs exhibited the multipotency characteristic of fetal MSCs into high passages. This study used cells from passages 18–35. These were cultured in a humidified incubator at 37 °C with 5% CO_2_ with complete culture basal media and expanded to 20 × 10^6^ cells per batch of microspheres.

### 2.2. Cell Encapsulation

hTERT-MSCs were microencapsulated as previously described [8,9]. Briefly, hydrogel prepolymer solution was prepared by combining 10 % *w/v* 10 kDa PEGDA (Laysan Bio, Arab, AL, USA), 37 mM 1-vinyl-2-pyrrolidinone, 1.5% *v/v* triethanolamine, 0.1 mM eosin Y, and 1% *w/v* Pluronic Acid F68 in HEPES buffered saline (pH 7.4). hTERT-MSCs were collected following incubation with trypsin, centrifuged at 300 g for 5 min, then resuspended in hydrogel prepolymer solution at 1 × 10^4^ cells/µL. A hydrophobic photoinitiator solution was prepared by combining 300 mg 2,2-dimethoxy-2-phenyl acetophenone in 1 mL 1-vinyl-2-pyrrolidinone. The hydrophobic photoinitiator solution was combined with sterile filtered mineral oil at 3 µL/mL. Prepolymer containing cells was injected into mineral oil solutions, vortexed for 2 s under white light (MH-100 metal halide lamp, Edmund Optics, Barrington, NJ, USA) and exposed to the white light for an additional 20 s to photopolymerize resulting emulsion droplets. Photopolymerized microspheres were washed twice with complete culture medium, centrifuged at 300 g for 5 min, and maintained in 6-well plates in complete basal culture medium in a humidified incubator at 37 °C with 5% CO_2_.

### 2.3. Osteodifferentiation and Calcein Staining of Encapsulated Cells

Osteogenic medium was prepared by adding the following to complete culture basal medium: 10 nM dexamethasone, 1 mM sodium glycerophosphate, and 50 µg mM l-ascorbate. Twenty-four hours following encapsulation, microspheres were separated into groups receiving basal medium or osteogenic medium supplemented with calcein (1 µg/mL). Medium was changed every 2 days.

### 2.4. Viability Assays

Parallel microencapsulated hTERT-hMSCs not incubated with calcein were evaluated for viability using an Ethidium homodimer-1/Calcein acetoxymethyl (Calcein AM) LIVE/DEAD^®^ Viability/Cytotoxicity Kit (Life Technologies, Carlsbad, CA, USA) on Days 3, 20, 34, and 64. Although calcein is not a cell-permeant fluorescent dye, calcein AM is membrane permeable and not fluorescent. Following entry into the cell, intracellular esterases cleave the AM ester group, resulting in the membrane-impermeable fluorescent calcein dye. Dead cells with compromised membranes cannot retain calcein. Ethidium homodimer-1 is membrane impermeable and has weak fluorescence until bound to DNA, which it accesses through the permeable membranes of dead cells. Microsphere samples (50 µL) from each time point were incubated in complete culture basal media containing calcein AM (2 mM), and ethidium homodimer-1 (4 mM) for 10 min in a humidified incubator with 5% CO_2_ at 37 °C before imaging with an epifluorescent microscope (Axio Observer ZI, Carl Zeiss, Inc., White Plains, NY, USA). Cells were observed at 10× magnification with 2 fluorescent channels to observe labeled live (green, ex/em; ~495/515 nm) and dead (red, ex/em; ~528/617 nm) hTERT-hMSCs. Hydrogel microspheres were imaged in triplicate for each green fluorescent, red, and phase contrast channels. Images were threshholded and counted using NIH Image J to obtain percentage viability as 100% × Live Cells ÷ (Live Cells + Dead Cells).

### 2.5. Alkaline Phosphatase Staining of Encapsulated Cells

Microencapsulated hTERT-hMSCs (50 µL samples) were stained on day 2 using a Fluorescence Alkaline Phosphatase Detection kit. To facilitate multiple washes without loss to aspiration, microspheres were placed in 40 µm mesh sterile cell strainers, that were transferred to successive solutions. Microspheres were first washed with phosphate buffered saline (PBS) containing TWEEN^®^ (PBST) pH 7.4, followed by 5 min incubation in fixation buffer, PBST wash, then 30 min incubation in staining solution. Stained microencapsulated hTERT-hMSCs were then rinsed, stored in PBS, and imaged in triplicate using brightfield color microscopy. Alkaline phosphatase positive cells stain purple. 

### 2.6. Alizarin Red S Staining of Encapsulated Cells

Microencapsulated hTERT-hMSCs (50 µL samples) were stained with Alizarin Red S stain on days 2, 7, 32, and 50 as previously described [8,10]. Microspheres were placed in 40 µm mesh sterile cell strainers to facilitate washes. Microsphere samples were first washed with PBS, incubated for 15 min in 10% *v/v* formaldehyde, washed with distilled water, incubated with agitation for 30 min with 40 mM Alizarin Red S solution, then washed four times with distilled water. Stained samples were imaged under brightfield color microscopy. To compare identical microspheres with calcein and alizarin red stain, 10 µL samples of microspheres were photopolymerized in place into a 20 µL film spread on a glass slide.

### 2.7. Statistical Analysis

Viability data were obtained in triplicate and reported as mean ± standard error. Analyses were conducted with Microsoft Excel.

## 3. Results

### 3.1. Cell Encapsulation

**Figure 1:**
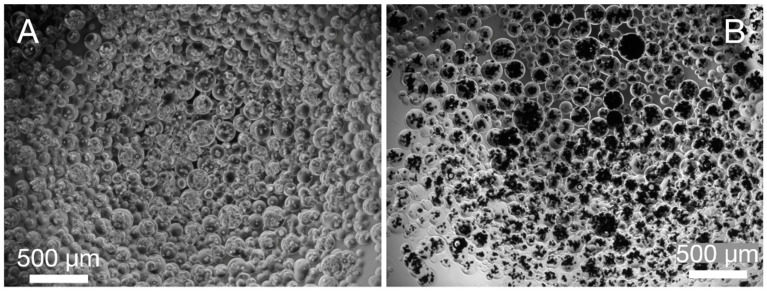
Phase contrast images hTERT-hMSCs at different time points following microencapsulation. Cells were imaged 24 h (**A**) and 3 weeks (**B**) after encapsulation using phase contrast microscopy. Cells appear bright and hydroxyapatite mineral deposits appear dark.

Upon microencapsulation, hTERT-hMSCs appeared bright within transparent microspheres when imaged using phase contrast microscopy. As cells began to mineralize hydroxyapatite, deposits appeared dark (Figure 1).

### 3.2. Cell Viability

**Figure 2:**
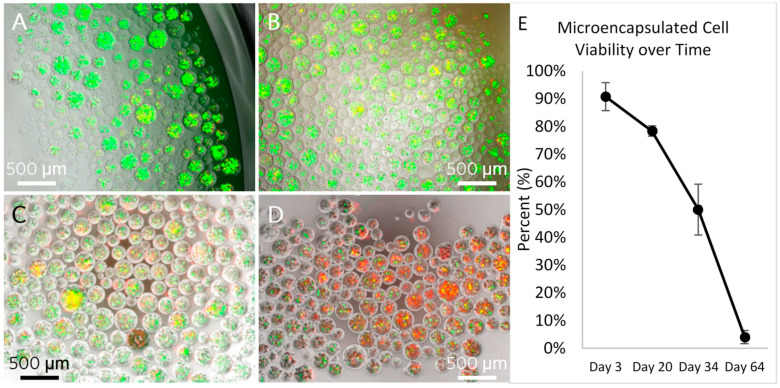
Calcium AM and ethidium homodimer-1 viability staining of microencapsulated hTERT-hMSCs over time. Green shows live and red shows dead. (**A**) Day 3, 90% ± 5% live cells. (**B**) Day 20, 78% ± 1% live cells. (**C**) Day 34, 50% ± 9% live cells. (**D**) Day 64, 4% ± 2% live cells. (**E**) Graph of viability over time.

Encapsulated hTERT-hMSCs began at 90% ± 5% viability on day 3 and decreased to 4% ± 2% viability on day 64. The live cells were stained green by cleaved calcein AM and dead cells were stained red by ethidium homodimer-1 dye (Figure 2).

### 3.3. Colorimetric Stains

**Figure 3:**
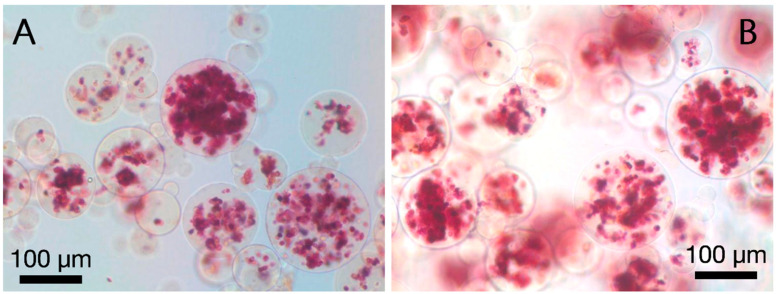
Alkaline phosphatase activity in basal maintenance medium (**A**) or osteogenic differentiation medium (**B**). There was no observable difference between groups.

Alkaline phosphatase activity as revealed by a purple stain was detected in both cells incubated in complete culture basal medium and cells incubated in osteogenic medium (Figure 3).

**Figure 4:**
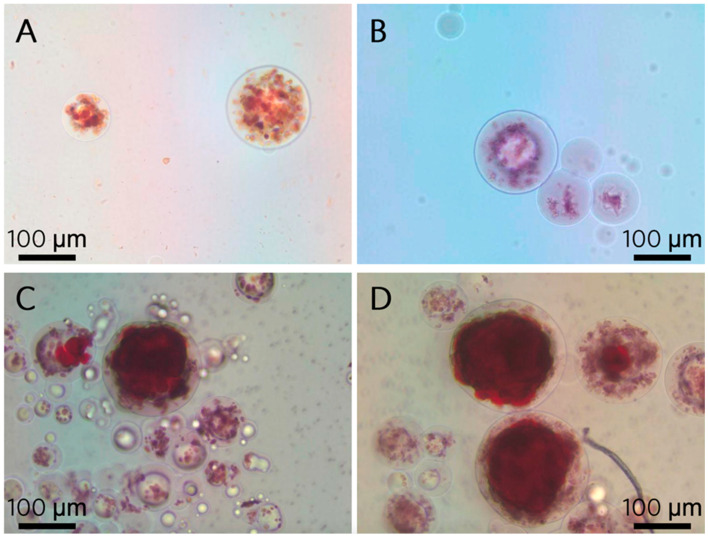
Representative Alizarin Red S stain of hydroxyapatite deposits at 2, 7, 32, and 50 days (**A**–**D**). (**A**–**C**) Complete maintenance basal media, (**D**) differentiation media. There were no observable differences between basal media and differentiation media, therefore (**A**–**C**) shows basal media.

Alizarin Red S stain was also detected in both microencapsulated hTERT-hMSCs receiving basal or osteogenic media (Figure 4). The onset of dark nodules characteristic of mineral stain occurred by 2 weeks. These nodules did not stain deep red until 4 weeks following microencapsulation.

**Figure 5:**
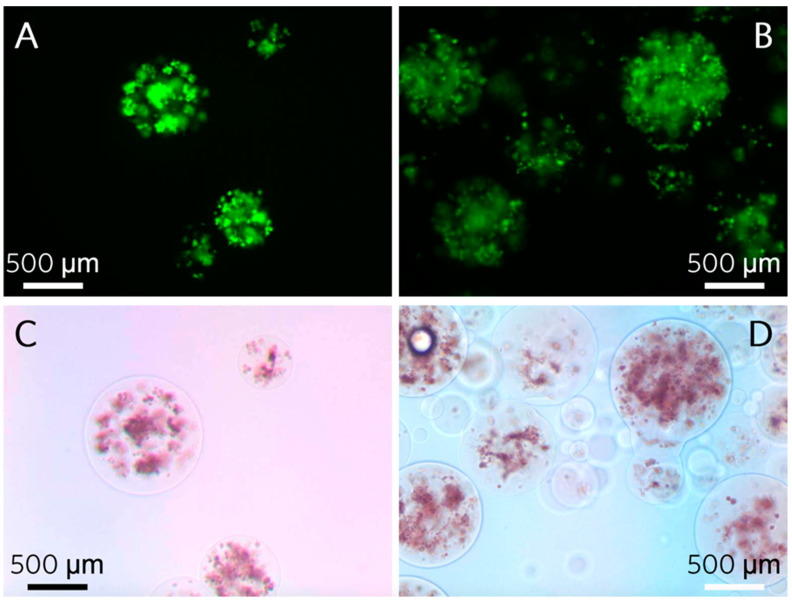
Two weeks post microencapsulation in basal maintenance (**A**,**C**) or osteogenic differentiation (**B**,**D**) media. Microencapsulated hTERT-hMSCs show fluorescent labeling of calcium in hydroxyapatite (**A**,**B**) and when identical microspheres were stained with Alizarin Red S, there was no red stain. There was no observable difference between microspheres receiving basal or osteogenic media.

Calcein stain within microspheres coincided with the appearance of dark nodules in phase contrast images, beginning at 2 weeks. Deep stain with Alizarin Red S did not appear until 4 weeks. Immobilized microspheres stained for both calcein and Alizarin Red S at 2 weeks show the former but not the latter stain (Figure 5).

## 4. Discussion

Assessing mineral deposits by microencapsulated cells can be challenging. Alizarin Red S stain is considered the gold standard, but the sensitivity of this stain to mineralization embedded within hydrogel microspheres has not been established. The goal of this investigation was to compare the efficacy of calcein and Alizarin Red S for assessing mineralization by microencapsulated osteoblasts. In addition to calcein’s advantages, in that it can stain living or fixed cells, our results demonstrate that calcein stains calcium minerals embedded within PEGDA hydrogel microspheres with a greater sensitivity than Alizarin Red S. Although calcein has been suggested as an alternative for Alizarin Red S stain for two decades, its adoption has not been widespread [1,2]. In monolayers and tissue sections, Alizarin Red S stain is effective, can be quantitated with acid-solubilization of calcium followed by measuring the dissolved stain, and does not require a fluorescent microscope. However, our results demonstrate that Alizarin Red S is not equally effective in microencapsulated cultures. As previous groups have dissociated calcein-stained monolayers to quantitate the stain using plate-readers, it may also be possible to similarly quantitate calcein-stained microencapsulated mineralization.

Although Alizarin Red S stain did not appear strongly in microencapsulated cultures less than 3 weeks old, an intense red stain was present in cultures 4 weeks or older. This may suggest a critical mass of calcium is required to stain red within PEGDA hydrogel microspheres. These mineral deposits could be observed under phase contrast microscopy, as dark opaque nodules that co-located with calcein stain. Additionally, alkaline phosphatase staining did not appear to be impacted by microencapsulation.

Finally, this study confirmed our previous observations that microencapsulation within PEGDA hydrogel microspheres is in itself osteoinductive [8]. Microspheres were polymerized such that entrapped cells were held in a rounded morphology, which has been reported to induce adipogenesis [11,12]. Nevertheless, these cells exhibited strong osteogenesis. We believe the osteogenesis is due to the similarity of our microspheres’ elastic modulus (100 ± 8 kPa) [13] to that of precalcified bone (100 kPa) [14].

The work herein describes the continuous staining of PEGDA-microencapsulated osteoprogenitors with calcein to assess their mineralization following differentiation. Although cells were encapsulated using 10 kDa PEGDA, the stain is broadly applicable to other materials used for cell microencapsulation. We previously established the mesh size of our 10 % 10 kDa PEGDA hydrogels to be 280 Å and that dextran molecules up to 20 kDa were readily released from these hydrogels [15]; the molecular weight of calcein is 622.55 g/mol. Considering that the mesh size of encapsulated cells must be large enough to permit nutrient and waste exchange as well as entry of serum proteins, it is likely that calcein could be used to assess mineralization in many cell microencapsulation systems. Further, although it is possible to label microencapsulated hydroxyapatite with Alizarin Red S stain, in early stages Alizarin Red S is not as sensitive as calcein stain, nor even as sensitive as phase contrast microscopy. This study demonstrates that in comparison to Alizarin Red S stain, calcein is better suited to monitor microencapsulated osteogenesis.

## Data Availability

Data is available upon reasonable request.
